# Protein–lipid complexes: molecular structure, current scenarios and mechanisms of cytotoxicity

**DOI:** 10.1039/c9ra07127j

**Published:** 2019-11-13

**Authors:** Esmail M. El-Fakharany, Elrashdy M. Redwan

**Affiliations:** Protein Research Department, Genetic Engineering and Biotechnology Research Institute, City for Scientific Research and Technology Applications (SRTA-City) New Borg EL-Arab 21934 Alexandria Egypt esmailelfakharany@yahoo.co.uk; Department of Biological Sciences, Faculty of Sciences, King Abdulaziz University P. O. Box 80203 Jeddah Saudi Arabia

## Abstract

Some natural proteins can be complexed with oleic acid (OA) to form an active protein–lipid formulation that can induce tumor-selective apoptosis. The first explored protein was human milk α-lactalbumin (α-LA), called HAMLET when composed with OA in antitumor form. Several groups have prepared active protein–lipid complexes using a variety of approaches, all of which depend on target protein destabilization or direct OA–protein incubation to alter pH to acid or alkaline condition. In addition to performing vital roles in inflammatory processes and immune responses, fatty acids can disturb different metabolic pathways and cellular signals. Therefore, the tumoricidal action of these complexes is related to OA rather than the protein that keeps OA in solution and acts as a vehicle for transferring OA molecules to tumor cells. However, other studies have suggested that the antitumor efficacy of these complexes was exerted by both protein and OA together. The potential is not limited to the anti-tumor activity of protein–lipid complexes but extends to other functions such as bactericidal activity. The protein shell enhances the solubility and stability of the bound fatty acid. These protein–lipid complexes are promising candidates for fighting various cancer types and managing bacterial and viral infections.

## Abstract 2

Some natural proteins can be complexed with oleic acid (OA) to form an active protein–lipid formulation that can induce tumor-selective apoptosis.

## Graphical abstract

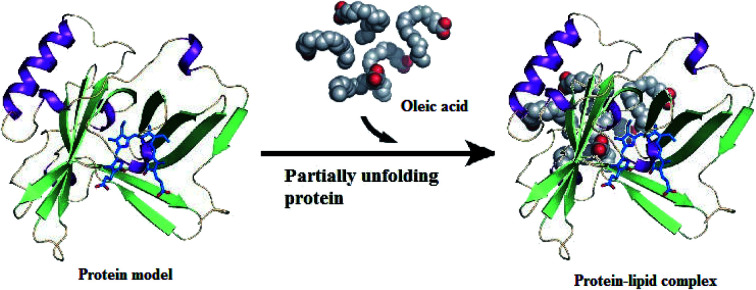

## Introduction

The protein–OA complex called HAMLET (Human Alpha-lactalbumin Made LEthal to Tumor cells) was described for the first time by Håkansson *et al.*^1^ while investigating the anti-adhesive properties of some human casein fractions. The human casein fraction which inhibited *Streptococcus pneumoniae* from adhering to respiratory epithelial cells was found to exhibit anticancer activity against numerous tumor cells *in vitro*. The HAMLET complex also exhibited significant selective apoptotic effects on cancer cells in both pre-clinical and clinical studies.^2–5^ Purified bovine or camel α-LA or other α-LA species can also bind OA and convert to complexes called BAMLET or CAMLET.^6,7^ In addition to the formation of a HAMLET complex using whole α-LA, small fragments produced by bovine α-LA proteolysis were able to bind OA and form complexes, called HAMLET-like complexes, with the ability to exhibit apoptotic activity against tumor cells.^8^ Other studies revealed that HAMLET-like complexes can be prepared using types of proteins other than α-LA.^6,9^ Lysozyme can bind OA and form lysozyme–OA complex with antitumor activity.^10^ Furthermore, β-lactoglobulin binds sodium oleate fatty acid and forms a HAMLET-like complex with cytotoxic activity against cancer cells.^11^ Similar to α-LA, lactoferrin (LF) efficiently binds OA and forms an LF–OA complex with 10 times more potent anticancer activity than α-LA–OA.^12^ Both α-LA and LF interacted with OA through hydrogen bonds and van der Waals forces, while bovine LF showed greater binding of fatty acid molecules than bovine α-LA.^12^ In addition, OA interacts with albumin isolated from different milk species and forms complexes with high antitumor activities against a variety of tumor cell lines.^13^ HAMLET and HAMLET-like complexes are of significant interest in research as promising tumor therapies. These complexes are tumor-selective drugs that effectively and specifically kill numerous tumor cells while avoiding normal healthy mature cells. Cancer treatment with chemotherapeutic drugs causes severe side effects, such as mucositis, anaemia, long-term neutropaenia, mutagenic changes, neuropathy, or chronic heart failure. Herein, we review different approaches to the preparation and formation of cytotoxic protein–lipid complexes and study their biological characteristics. We describe the conformational characteristics of protein–lipid complexes and their activities in both *in vitro* and *in vivo* studies of cancer cells. We also review other biological activities of these active protein–lipid complexes in addition to their different anticancer mechanisms.

## Different approaches for preparation of the cytotoxic protein–lipid complexes

**Fig. 1:**
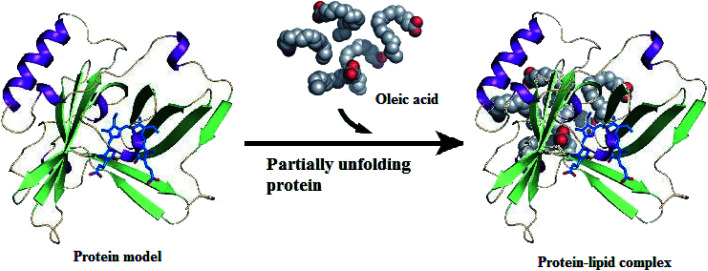
Protein–lipid complex formation. Protein is partially unfolded and binds to fatty acids to form protein–lipid complex. Structure of oleic acid is obtained from Protein Data Bank ID: 1GNI.

Preparation of active protein–lipid complexes by different research groups has significantly increased. It has been reported that the protein conformation induced by release of ions at an acidic pH favors the chelation of OA, such as release of Ca^2+^ from α-LA at an acidic pH^14^ and release of bound ions from LF at an acidic pH,^12^ in order to obtain a more open protein structure.^15^ In cytotoxic protein–lipid complexes, the protein is at first assumed to become destabilized in an unfolded conformation, then it can bind OA in numerous stoichiometries (Fig. 1). The following is an overview of the different ways to prepare cytotoxic protein–lipid complexes, as summarized in Table 1.

**Table 1:** Common approaches to prepare the active protein/OA complexes

Procedure type	Protein	Procedure dependence	Protein/OA molar ratio	Ref.
Column chromatography	Human α-LA, bovine α-LA, equine lysozyme	Depends on removing Ca^2+^ from protein with EDTA bound to column pre-conditioned with OA	1/∼1–5 for α-LA/OA, 1/∼5–35 for equine lysozyme/OA	16–18, 33, 35, 37 and 48
Alternative method	Bovine α-LA	Depends on partially heated holo protein followed by loading to OA pre-conditioned column	Not assayed	11
Heat-induced method	Camel, human and bovine α-LA, bovine LF, bLG, camel, human and bovine albumin	Exposure of holo form of protein to heating at 50–60 °C, then add OA directly	1/∼2.5–10 for α-LA/OA and bLG/OA, 1/15.8 for bovine albumin/OA, 1/12.9 for human albumin/OA, 1/17.9 for camel albumin/OA	7, 11–13, 19 and 48
Direct mixing method	α-LA, parvalbumin, bLG	Direct incubation of protein with OA at change in pH to acid or alkaline environment	1/4.5 for OA/α-LA, 1/13 for OA/parvalbumin, 1/17 for OA/bLG	8, 20, 21 and 23–25

### a. The anion exchange chromatographic approach

HAMLET was initially prepared by loading the apo-form of human α-LA on a DEAE-Trisacryl Manion column (exchange chromatography) pre-conditioned with OA and equilibrated at pH 8.5.^16^ This method is conventional for converting α-LA to toxic form, after elution of the cytotoxic protein–OA complex from the exchange chromatographic column by a gradient of NaCl solution up to 1.5 M. Svanborg and colleagues used only this conventional method to prepare HAMLET complex from the apo form of α-LA.^17^ This method depends on the release of Ca^2+^ from the purified native holo form of α-LA through EDTA treatment and hydrophobic interaction chromatography, subsequent to partial unfolding of α-LA.^16^ Brinkmann *et al.* used a modified method to prepare HAMLET and BAMLET, using an anion exchange chromatographic DEAE-Sepharose column instead of a DEAE-Trisacryl Manion column.^18^ The column matrix resin was mixed with fatty acid (OA) at alkaline pH (pH 8.5), then exposed to sonication and strong mixing, followed by packing this OA/resin into the column instead of the pre-condition of the packed column with buffer containing OA. Because of the OA dispersion throughout the column, this modified method is expected to be more efficient than the conventional method of protein interaction with OA by converting it into a protein–OA complex. Moreover, this method produces a high yield of active protein–OA complexes. Other alternative methods have been used, including a partially heated holo protein loaded to an OA pre-conditioned column.^11^

### b. Heat-induced changes of protein approach

In the column approach, HAMLET was formed by destabilizing α-LA by releasing Ca^2+^ bound with EDTA, followed by application to a pre-conditioned anion exchange chromatographic column with the fatty acid and elution from the column by a gradient of salt solution. The method of heat-induced change depends on the breakdown of protein disulfide bonds and the binding of free reactive thiol groups.^15^ In general, it was found that the exposure of the holo form of protein to heating at 50–60 °C was sufficient to unfold the protein and favored the binding of OA, even without the use of a calcium chelator such as EDTA.^19^ The tumoricidal activity of the active protein–lipid complexes resulting from this method was analogous to that of the prepared exchange columns regardless of the preparation method. In recent studies, heat-induced changes in human, bovine and camel albumens and camel α-LA showed some similarities to those of human and bovine α-LA.^7,13^ Bovine LF was also heat treated and found to be capable of binding OA about two-fold greater than α-LA.^12^

### c. Direct mixing approach

Direct mixing of protein and OA at room temperature with exchanged pH environments has been used to form active protein–OA complexes.^8,20,21^ However, Svanborg and colleagues established that direct binding of OA to protein is not sufficient to form a successful cytotoxic active protein–OA complex.^16,22^ Fang *et al.*^23^ demonstrated that the cytotoxic α-LA–OA complex was formed by directly adding OA to dissolved 0.7 mM of protein in citric acid–phosphate buffer with different pH values ranging from 2.0 to 9.0 at a molar ratio of 1 : 50 protein/OA. It was found that, in the pH values ranging from 7.0 to 9.0, α-LA exposure formed active protein–OA complexes with higher cytotoxic efficacy. Moreover, Kehoe and Brodkorb^24^ revealed that cytotoxic protein–OA complexes could be formed by mixing protein with OA in high shear environments. It was found that mixing the dissolved protein in Tris–HCl, pH 7.4, at concentrations of 10–15 mg with dissolved ethanol OA, followed by mixing vigorously with a vortex, reaches a final protein/OA molar ratio of 1 : 10 or 1 : 15 and results in a complex that can be used in tumor killing. The shear method has been used to prepare active protein–OA complexes of numerous proteins other than α-LA, such as human serum albumin, bovine β-lactoglobulin and maltose binding protein. In addition to OA, cytotoxic protein–lipid complexes can be formed by binding to other long-chain unsaturated fatty acids, including palmitoleic, vaccenic and linoleic acids, and results have shown that the complexes of bovine α-LA prepared with palmitoleic and linoleic acids were more effective against tumor cells than those prepared with OA.^24^ In addition, direct mixing of native protein and fatty acid followed by exposure to alkaline conditions was used to prepare bioactive complexes.^25^ A cytotoxic protein–OA complex was prepared by incubating 0.6 mM of bovine α-LA at 45 °C and pH 12.0 with ethanol solution containing 22 mM OA. Spolaore *et al.*^20^ were able to prepare the BAMLET complex by direct mixing of the protein with OA followed by loading the protein/OA mixture to a Sephadex G-150 column (size exclusion chromatographic column).

## The biological properties of the cytotoxic protein–OA complexes

The successful formation and stabilization of cytotoxic protein–OA complexes was found to require mutual partial unfolding of the protein and exposure of OA as a co-factor.^16,26^ It has been exhibited that the antitumor effects of HAMLET and HAMLET-like complexes are mainly independent of the C-terminal portion of the protein and the β-sheet domain. The partial unfolding of the protein through metal ion chelation by EDTA or heat treatment leads to destabilization of the β-sheet domain and leaves the α-helix domain unaffected, which favors fatty acid binding and thus creates cytotoxic complexes such as HAMLET.^16,27^ In agreement with this, the separated α-helix domain of human α-LA was capable of binding OA and forming a cytotoxic protein–OA complex.^28^ Human α-LA in HAMLET initially exhibited an oligomeric form, so α-LA was called MAL (multimeric alpha lactalbumin).^29^ MAL was purified from human skim milk using a DEAE-Trisacryl M chromatographic column followed by size exclusion column chromatography. By contrast, the Lund research group assumed that α-LA in HAMLET was in a monomeric form, stating that “HAMLET is mostly in a monomeric protein”^30,31^ or “homogeneously monomeric”.^27^ As a result, the loss of protein tertiary structure but retention of secondary structure favors a steady intermediate fold and formulates a molten globule. In addition, variations in topology characteristics compared to the native state were also identified by partial hydrolysis.^27^ In the HAMLET complex, α-LA retains its partially unfolded characteristics even under physiological salt circumstances,^32,33^ implying that the OA stabilizes the protein in a partially unfolded form.

**Fig. 2:**
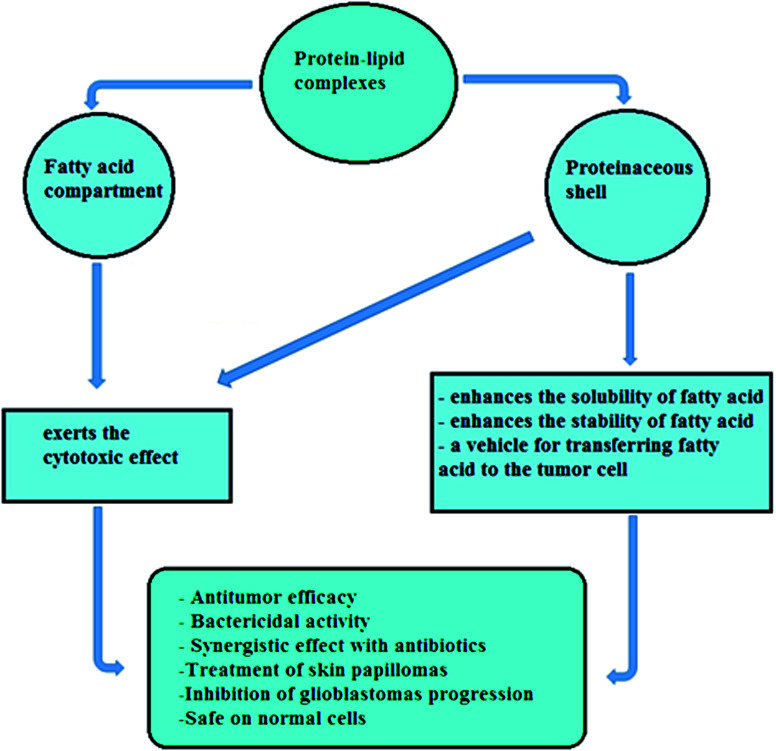
Overview of multiple biological functions of protein–lipid complexes. These biological functions of protein–lipid complexes are exerted by the fatty acid compartment rather than by proteinaceous shell or both protein and fatty acid together.

Alteration in the protein conformation might induce the formulation of HAMLET-like complexes. Tolin *et al.* revealed that the entire 123-residue sequence of α-LA was not required for tumoricidal efficacy, as peptide fragments of α-LA were found to be able to induce apoptosis in Jurkat tumor cells after binding with OA.^8^ Moreover, it was found that recombinant human α-LA modified with alanine residues instead of cysteine residues could exert cytotoxic action similar to the HAMLET complex.^34^ Additionally, mutant α-LA with no Ca^2+^ binding activity has also been converted to cytotoxic complexes with OA after shifting Asp87 to alanine, suggesting that a functional Ca^2+^ binding site is not essential for the conversion of α-LA to the tumoricidal complex.^35^ In contrast, structural changes caused by calcium binding do not affect the tumoricidal efficacy of the formed bioactive complex.^35,36^ The HAMLET complex also showed a high calcium binding affinity in physiological salt circumstances with a calcium constant of 5.3 × 10^6^ M^−1^.^35^ It was found that the exposure of native α-LA, apo-α-LA or partially unfolded α-LA alone does not show any tumoricidal activity, irrespective of the native camel α-LA, apoptosis was found in cancer cells as revealed in recent study by Uversky *et al.*^7^ These results have led scientists to focus on the likelihood that the tumoricidal activity of cytotoxic protein–OA complexes depends on the OA component and the protein component independently (Fig. 2).^16,27,35,37^ The high variability in the protein moiety in these complexes strongly suggests that the antitumor efficacy is due to OA, while the protein has no cytotoxic effect on tumor cells in its native state.^8,38^ However in other studies, the tumoricidal activity of protein–OA complexes has been caused by both protein and OA, as in the cases of bovine LF^12^ and bovine, human and camel albumins.^13^ It has therefore been suggested that the protein is only a carrier of OA or a synergistic factor in tumor activity with bound OA.^22^ In addition, the antitumor activity of other proteins formulated with OA, such as β-lactoglobulin and paralbumin complexes, was found to be similar to HAMLET's, indicating that the protein is less important than the OA component in association with tumoricidal activity (Fig. 2).^39^ As extensively reported,^40^ the HAMLET, BAMLET or – like structures are also deactivated by many agents, such as bovine serum albumin, fetal calf serum, and calcium.

## Conformational features of the cytotoxic protein–OA complexes

Human α-LA protein is the first example of a protein that shows a distinct function in its native form and can be converted to a new form with a bioactive role after partial unfolding and binding with OA to form the HAMLET complex.^33^ It has been suggested that the alteration in protein folding leads to changes in tissue environments, which can allow a single polypeptide chain to play highly diverse and positive biological roles in different tissue sections.^16^ The low resolution small angle X-ray scattering (SAXS) conformational structure of HAMLET showed a two-domain conformational structure with a stretched C-terminal domain and a large globular domain. The SAXS structure also established a HAMLET molecular weight of 15 ± 2 kDa.^41^ Furthermore, the results obtained from investigation of HAMLET complex conformation through tryptophan fluorescence spectroscopy and both remote and near-UV CD spectroscopy showed that the active complex maintains a partially unfolded, stable and molten globular structure in physical environments, unlike partially unfolded α-LA which returns to its native state in normal physiological conditions.^22,35,37,42,43^

It was suggested that active protein–OA complexes consisting of protein formulated with fatty acids, such as BAMLET, CAMLET, lysozyme–OA, β-lactoglobulin–OA or pike parvalbumin–OA complexes, should be able to form cytotoxic protein–OA complexes as a result of partial protein development.^44^ There are large variations in stoichiometry and size of protein–lipid fatty acids as a result of the different ways of binding fatty acids to proteins and the multiple protein formulation approaches with OA. Therefore, one bioactive protein–OA complex with a single stoichiometry or defined mass weight does not exist. In addition, some toxic proteins have been found to form cytotoxic complexes in neurodegenerative diseases after binding with long-chain unsaturated fatty acids such as OA and arachidonic acid. For instance, alteration in superoxide dismutase by mutation was found to formulate new cytotoxic granular aggregates with OA and arachidonic acid.^45^ Furthermore, it was found that mutant α-LA with Ala substituted for eight Cys residues was able to form a molten globule conformation in physiological environments, leading to an overall yield of HAMLET and actually increasing the stoichiometry of the α-LA–OA complex.^34^ These findings suggest that the ability to prepare these cytotoxic protein–lipid complexes is a common feature of the polypeptide chain structure, as is the ability to form granular aggregates. These findings also include the right conditions for their formulation and effectively turning any native folded protein to favor OA.^38^

On the other hand, there is much focus on how fatty acids affect protein properties and conformations. Initially, reports emphasized the significant role of OA. It has been shown by NMR investigation that OA adopted a monomeric α-LA state in a compact structure with a predominance of >95%.^22^ Complexes featuring oleic acid (OA) with human α-lactalbumin, bovine α-lactalbumin or human lactoferrin were recently investigated using small-angle neutron scattering (SANS). It was shown that, while α-lactalbumin protein complexes formed on the surface of polydisperse OA micelles of various sizes (>10–100 nm), the LF complexes comprised a monodisperse system of small (∼20 nm) particles.^46^ The study did not reveal any ratio competition entry into HeLa cell nuclei between the two kinds of protein complexes based on their nano sizes. There was also no cytotoxicity for the formed complexes when OA was substituted with saturated fatty acids with 14–18 carbon atoms.^16^ Svensson *et al.* implied that the stereo-specific interaction between α-LA and OA was found to favor *cis*-specific conformation.^35^ However, another study successfully developed cytotoxic complexes with *trans* fatty acids like elaidic acid and stearic acid.^47^ In addition, Brinkmann *et al.* revealed that protein-fatty acid complexes formed with cytotoxic activity against tumor cells regardless of whether the fatty acids were saturated or unsaturated or *cis* or *trans* in structure, since each formed complex had a toxicity level.^47^ The active complexes with unsaturated fatty acids, such as OA, linoleic acid, vaccenic acid, elaidic acid and palmitoleic acid, have been found to have higher cytotoxic effects than the complexes formed with saturated fatty acids such as stearic acid.

## Activities of the cytotoxic protein–OA against tumor cells *in vitro*

There are numerous proteins capable of binding OA to form bioactive protein–OA complexes with significant selective tumoricidal efficacy against a number of tumor cell lines. Despite the selectivity of cytotoxic protein–lipid complexes towards tumor cells remaining undefined, numerous *in vitro* studies have shown that these complexes may have potential therapeutic efficacy. HAMLET complex showed cytotoxic efficacy against various tumor cell lines, including colon cancer cells, bladder cancer cells and human glioblastoma xenograft cells.^44^ In addition to the HAMLET complex's ability to kill different tumors and undifferentiated cells, many studies have found the same killing efficacy of BAMLET complex on both cancer cells and normal primary cells.^18^ Brinkmann *et al.* also revealed that primary endothelial cells were found to be the most resistant to BAMLET complex, while primary peripheral mononuclear blood cells were found to be the most sensitive cells when freshly separated from human blood.^18^ Additionally, BAMLET complex can cause lysis of erythrocytes.^18^ The LC50 (the concentration required to kill 50% of the cells) values of BAMLET and HAMLET seemed to be similar in tumor cells.^22,47^ There are also similarities in morphology, uptake and terminal deoxynucleotidyl transferase dUTP nick-end labeling of HAMLET and BAMLET complexes on A549 lung tumor cells, in addition to the initiation of necrosis-like cell killing in THP1 cells in both complexes. Furthermore, HAMLET complex was found to be similar to BAMLET complex in sensitivity against primary human vascular smooth muscle cells and induced cell death.^48^

Later, similar antitumor efficacy was exerted by OA exposed to different α-LA types purified from skim milk of mammalian species other than human and bovine, including goat and camel, forming GAMLET and CAMLET complexes (for goat or camel α-LA made lethal to tumor cells), respectively.^7,49^ The CAMLET complex showed strong tumoricidal activity against the Caco-2 colon tumor cell line, PC-3 prostate tumor cell line, HepG-2 hepatoma cell line and MCF-7 breast carcinoma cell line.^7^ Furthermore, the antitumor activities of OA bound to other proteins, like pike parvalbumin and bovine β-lactoglobulin, were analyzed.^38^ This experimental analysis showed that the formed active OA–protein complexes were cytotoxic against HEp-2 tumor cells and the *S. pneumoniae* D39 cell line and the cytotoxicity of these active complexes was associated only with the fatty acid (OA) part of the complex, not with the proteinaceous shell.^38^ Additionally, apo-myoglobin, β2-microglobulin and canine milk lysozyme were all capable of forming cytotoxic protein–OA complexes after treatment of the protein with OA in physiological conditions, where their molten globular conformation remained stable, and all the prepared complexes showed a HAMLET-like tumoricidal effect on L1210 leukaemia cells.^49^ All these results suggest that the protein component of the bioactive protein–lipid forms is not the source of any cytotoxic effect, but the action of the OA is the source of antitumor effects and the protein moiety works as a carrier of OA or other fatty acid molecules through the cell membrane into cancer cells, in addition to enhancing the solubility of these toxic fatty acids (Fig. 2).^17,49^

Chemotherapy-resistance of pleural mesothelioma ATP synthase was reduced after BAMLET exposure in regular use. This is in line with the Ho *et al.* study^50^ in which they revealed a direct effect of HAMLET on ATP synthase in a dose-dependent reduction in cellular ATP levels detected in lung carcinoma cells, where HAMLET was found to be colocalizing with the nucleotide-binding subunit and the catalytic units α and β of F_1_F_0_ ATP synthase.^50,51^ BLAGLET and BAMLET increased the cytotoxicity in mesothelioma by holding increasing amounts of oleic acid in an active state encapsulated in increasingly unfolded protein components, although BAMLET formed rounded aggregates while BLAGLET formed longer fiber-like aggregates.^52^ This aggregation may be due to the protein component tail model,^53^ where spherical droplets of oleic acid are encapsulated by the partially unfolded protein component and the non-associated component can interact with other BAMLET molecules' tails.^54^ Recently, in contrast to the above tumor activities, various albumins purified from camel, human and bovine milk have shown powerful antitumor activity by both OA and protein shell. The binding of OA to albumins led to the development of active OA–albumin complexes with significant effectiveness against HepG-2, Caco-2, MCF-7, and PC-3 cancer cell lines.^13^ El-Fakharany *et al.*^13^ revealed that the cytotoxic camel albumin–OA complex exhibited noticeable tumoricidal effects by both protein shell and OA component. These findings were consistent with the results of Fang *et al.*,^12^ who revealed that bovine LF bound to OA and formed an active LF–OA complex with potent tumoricidal activity against HT29, MCF-7 and HepG2 cells. Bovine LF had a strong fatty acid affinity with bovine α-LA, although both LF and α-LA interacted with OA through hydrogen bonds and van der Waals forces.^12^ They also demonstrated that the antitumor activity of LF–OA complex was exerted by both protein shell and OA component (Fig. 2).^12^

## Activities of the cytotoxic protein–OA against tumor cells *in vivo*

A main object when investigating the cytotoxic efficacy of protein–OA complexes is apparent specificity and selectivity against tumor cells, sparing the normal cells. As a consequence, for *in vitro* studies, scientists highlighted the role of HAMLET and HAMLET-like complexes *in vivo* in possible applications for cancer treatment.^3–5,55,56^ There is one limitation in using HAMLET and HAMLET-like complexes as potential therapy for cancer, which is that these complexes interact with albumin. One function of albumin is carrying and solubilizing the long chain fatty acids which allow the binding of free fatty acids, as in HAMLET and HAMLET-like complexes, neutralizing the cytotoxic activity of these complexes.^4,18,57^ Also, due to the existence of albumin in most physiological fluids, HAMLET and HAMLET-like complexes will have difficulty reaching the target tumor cells in a cytotoxic form. Mossberg *et al.* demonstrated that the local instillation of HAMLET complex into mice with bladder tumor stimulated rapid tumor progress reduction.^5^ Investigation by fluorescence imaging revealed that HAMLET complex was retained in mice with carcinoma compared to healthy mice. Moreover, HAMLET complex could decrease the tumor size in mice after five intravesical treatments; it significantly delayed cancer progress in mice with tumor compared to the control group of mice treated with free α-lactalbumin.^5^ In addition, intravesical treatment with HAMLET of 9 male patients with superficial bladder tumor induced apoptotic response in 6 out of 9 patients and induced rapid progress in the daily flaking of the killed cancer cells into the urine in 8 of 9 tumor patients. Also, a decrease in tumor mass and an alteration in tumor feature were detected at surgery in 8 of 9 patients.^4^ Fischer *et al.* also demonstrated that the HAMLET complex had a potent therapeutic efficacy on glioblastoma multiforme disease.^2^ Treatment was performed after xenotransplantation of human glioblastoma biopsy spheroids into rat^nu/nu^ brains. The active HAMLET complex penetrated to the solid tumor and stimulated apoptosis in tumor cells. HAMLET has been successful in delaying both tumor progress and persistence and significantly reducing the abnormal tissues in the brain.^2,58^ Moreover, the tumoricidal efficacy of HAMLET against colon tumor was examined in a model of human with colon tumor (APC^Min^/þ mice). This model's progress in intestinal cancer is considered a human model. Oral administration of HAMLET complex led to decrease in tumor mass and in the number of polyps in this APC model, which caused a potent decrease in tumor mass.^59^ In addition, it was found that HAMLET complex accumulated in solid tumors. The expression of oncoprotein markers such as β-catenin, COX, VEGF and 2Ki67 was found to be reduced after oral administration of HAMLET complex. It was also found that the HAMLET complex has an apoptotic effect in the mammary gland. Apoptotic cell killing in the mammary gland has been improved by the introduction of HAMLET complex in the mammary glands of lactating mice.^55^

## Antitumor mechanisms of the cytotoxic protein–lipid complexes

**Fig. 3:**
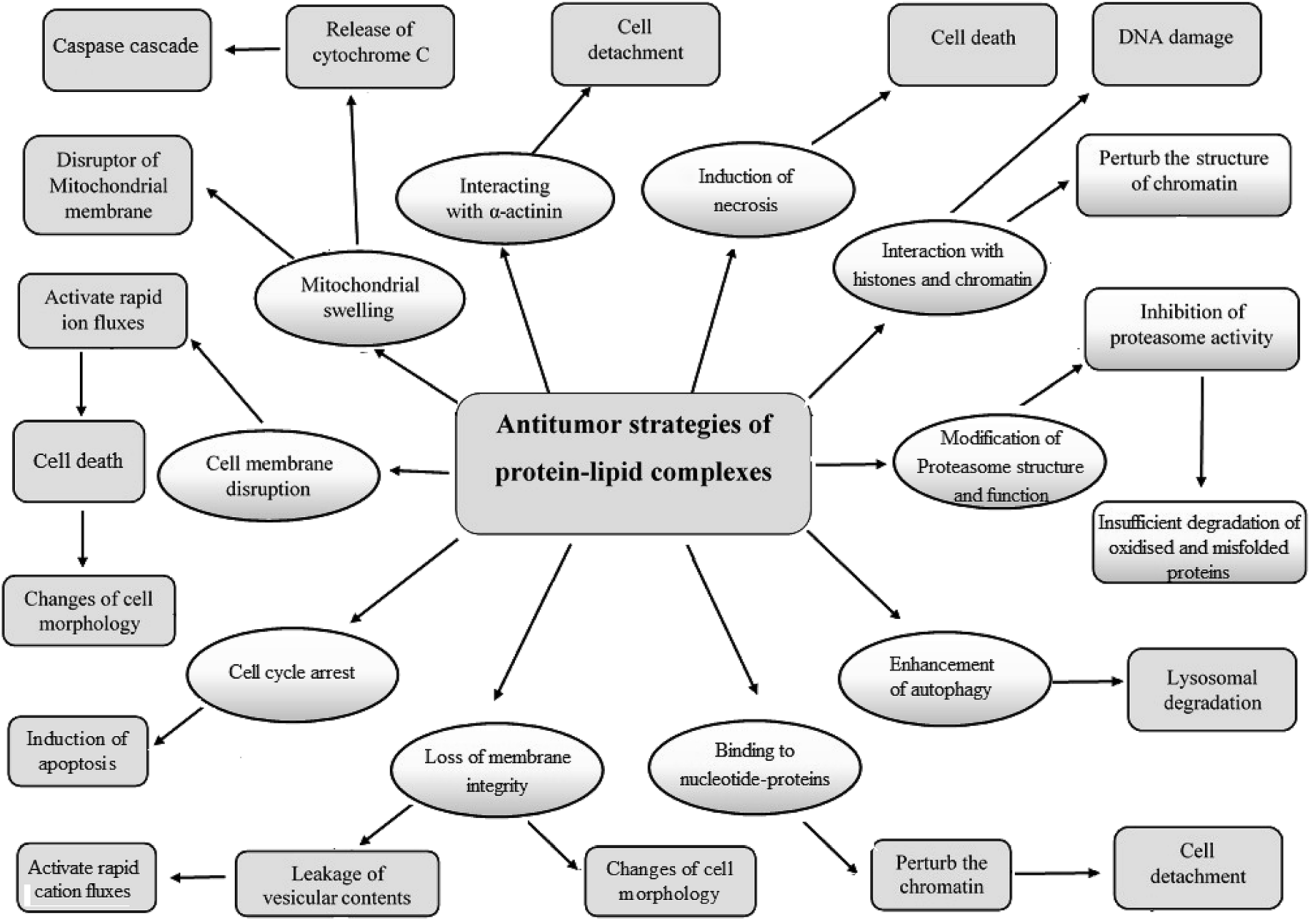
Suggested mechanisms and strategies of the cytotoxic protein–lipid complexes against tumor cells during cell death induction.

The protein–OA complexes are considered a potential antitumor agent in both *in vitro* and *in vivo* because of their ability to definitely penetrate tumor cells. These active complexes can trigger numerous mechanisms of cell death (Fig. 3 and Table 2) by affecting mitochondria, nucleosomes, endoplasmic reticulum, and proteasomes.^26,31,32^ Although the tumoricidal activity of the cytotoxic protein–OA complexes was associated with various pathways, including induction of apoptosis, binding to α-actinin to stimulate detachment of tumor cells or autophagy,^13,16,29,60–62^ motivation of ion channels in tumor cells causing a non-selective cation current^63^ which consequently effects the expression of mitochondrial F-ATP synthase, causing protein–OA dose-dependent decrease in the levels of intracellular ATP,^50^ alterations in proteasome structure,^64^ action on c-Myc and interruption of glycolysis by a precise effect on the HK1 glycolytic enzyme,^65^ interference with chromatin, histones and numerous nucleotide-binding proteins like kinases, GTPases, and ATPases,^66,67^ interface with lipid membranes,^32^ and the presence of constructive effects of the misfolded or partially folded proteinaceous part of the cytotoxic protein–OA complexes.^30,33^ Fang *et al.* recently developed a theoretical basis for clarifying the complex internalization of α-LA–OA.^68^ They showed that the α-LA–OA complex entered tumor cells similarly to HAMLET in a dose- and time-dependent manner. The internalization of α-LA–OA was dependent on the temperature and its anti-tumor activity was closely linked to the pathway of phagocytosis. Both the protein and the fatty acid components played an important role in internalizing α-LA–OA: the OA component was responsible for anti-tumor activity while the α-LA component could initiate certain specific targets or pathways. In parallel, the effect of lipids on host cells is controlled by the protonation state. The fatty acids can exert some their essential effects similar to HAMLET on host cells when in the deprotonated state and when presented in the context of a partially unfolded protein.^69^

**Table 2:** The tumoricidal strategies and mechanisms of the cytotoxic protein–lipid complexes

Protein–lipid complex	Strategies	Effects on tumor cells	Ref.
HAMLET	Apoptotic action	Enhances p53-independent apoptotic pathway or induces apoptotic-like cell death	18, 60 and 80
	Autophagy process	Enhances a macroautophagic mechanism	80 and 100
	Chromatin structure perturbation	Accumulates in the nuclei of tumor cells and interacts with histones, independent of the histone tail, leading to perturbation of chromatin structure and enhancing cell detachment	60, 66 and 92
	Cell detachment	Induces chromatin condensation through caspase-dependent and -independent stimulation	1 and 4
		Effects chromatin acetylation and exerts activity in synergy with histone deacetylase inhibitors	
		Binds to α-actinin and stimulates tumor cell detachment	
	Proteasome inhibition	Binds to 20S proteasome core, triggering conformational modifications and enhancing inhibition of its activity	44, 64, 93 and 94
	Apoptosis-promoting p38 pathway	Showed to be the top-scoring cell death mechanism	104
	Ribosome interactions	Interferes with individual ribosomes and intact ribosome proteins	
	c-Myc oncogene status	Increased expression of c-Myc oncogene enhances tumor cell death	65
BAMLET	Apoptotic action	Induces tumor cell death according to type of tumor cells	148
	Lysosomal mechanism	Triggers a caspase-independent lysosomal mechanism in tumor cells causing lysosomal membrane perturbation	84
Equine lysozyme–OA complex	Cellular membrane perturbation	Significant structural alterations upon interaction with lipid membranes	76
Bovine LF–OA complex	Apoptotic action	Induces apoptosis through both mitochondrial-mediated and death receptor pathways	12
CAMLET	Cell cycle arrest	Inhibits tyrosine kinase activity	7
	Apoptotic action	Inhibits tyrosine kinase activity and disrupts tumor cell signaling pathways	7
Albumin–OA complex	Cell cycle arrest	Causes induction of cell cycle arrest in a dose-dependent manner	13
	Loss of membrane integrity	Causes increase in the OA exposure, which leads to modification of the selective permeability of the cellular membrane	13
Bovine lactoglobulin–OA complex	Apoptotic action	Induces cell death mechanisms analogous to those of HAMLET	149
Pike pervalbumin–OA complex	Apoptotic action	Induces cell death mechanisms similar to those of HAMLET	150
Lactoglobulin–linoleic acid complex	Apoptotic action	Induces cell death mechanisms similar to those of HAMLET	151
rRecombinant His-tagged HAMLET	Apoptotic action	Activation of caspase-8 dependent on the autophagy-related proteins	152

### a. Actions on cellular membrane

**Fig. 4:**
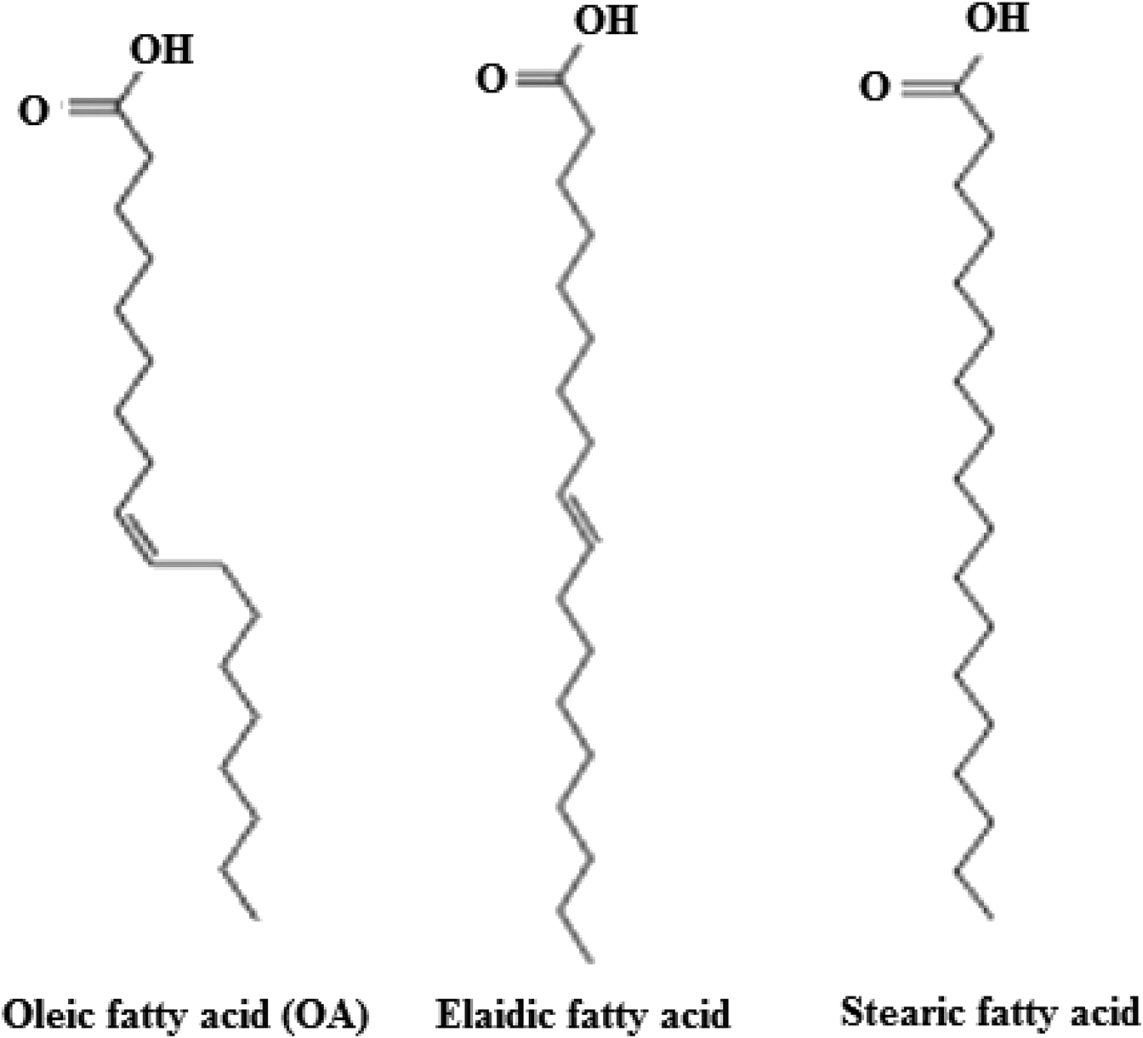
Structures of oleic, elaidic and stearic acids. OA (*cis*-9-octadecenoic acid, 18 : 1 *cis* Δ9) has a boomerang-like shape due to *cis* double bond that restricts the mobility between C9 and C10. Both elaidic acid (*trans*-9-octadecenoic acid, 18 : 1 *trans* Δ9, *trans*-isomer of OA) and stearic acid (octadecanoic acid, 18 : 1) have a rod-like shape.

The possible interpretation of the antitumor effects of the protein–lipid complexes emphasizes their effects on lipid cellular membranes and consequently on their permeabilization. The apo-form of α-LA has a higher binding activity to lipid bilayers than the holo-form^70^ and thus allows better interaction of the protein shell with the cell membrane. Because fatty acids in protein–lipid complexes seem to prevent relapse of proteins like α-LA to the holo-form, the exposure of hydrophobic areas is preserved, supporting protein–lipid complex surface affinity over the native protein. Moreover, the existence of an OA component in protein–lipid complexes such as HAMLET may increase affinity to the cellular membrane due to the structure of the aliphatic chain of OA, provided that this is not entirely seized by the protein.^71^ On the other hand, numerous biological properties of fatty acids have been associated with a physicochemical perturbation of the cellular membrane. *Cis*-unsaturated fatty acids like OA are markedly angular, with a boomerang-shaped “twist” in the structure, while *trans*-unsaturated fatty acids, like elaidic acid, and *trans*-saturated fatty acids, like stearic acids, have a linear structure (Fig. 4).^72^ This difference in molecular structure seems to have imperative biological and biophysical significance. The unbound *cis*-fatty acids bind to and interact with the cell membrane, leading to major structural changes in the lipid bilayer's hydrophobic core and causing membrane disruption.^73^ OA definitely exerts activity on cellular physiology, moderating some main cellular pathways such as signal transduction and inhibiting cellular processes like cell adhesion, secretion and division.^72^ It has been determined that the binding of cytotoxic protein–lipid complexes, such as HAMLET and equine lysozyme–OA complexes, alters the morphological characteristics of the cell membrane and affects its integrity, suggesting that membrane disruption may be a primary cause in enhancing cell death.^32^

HAMLET complex has been found to interact with negatively-charged and neutral giant and large unilamellar vesicles in neutral conditions and to perturb negatively-charged large unilamellar vesicles at pH 7.0 without any indication of uptake in the giant unilamellar vesicles. HAMLET complex has an effect on the integrity and permeability of the membrane in live cells, whereas α-LA has a weak affinity for the surface of the cell membrane with little to no cytoplasmic existence.^32,74,75^ Equine lysozyme–OA complexes interact with phospholipid membranes of giant unilamellar vesicles and supported bilayers with no significant effect on membrane permeability.^76^ After binding to phospholipid, the equine lysozyme–OA complex recovers some of its enzyme-native conformation activity, which causes OA to be released from the complex to the cellular membrane. Therefore, equine lysozyme is allowed to re-fold to a native conformation.^76^ In an experimental study using PC12 cells (single rat adrenal pheochromocytoma) to demonstrate accumulation of equine lysozyme–OA complex at the plasma membrane, the membrane was perturbed and the complex quickly internalized and filled the cell after 1 h, as determined by live microscopy.^77^ All of the examined protein–lipid complexes have been found to have a significant effect on lipid membranes, consequently enhancing membrane modifications as a possible description for the initiation of cell death.^71^

### b. Actions on GTPases and kinases

HAMLET complex effects the ion flux of tumor cells, causing a rapid activation of p38 MAPK response, as exposed by transcriptomic analysis.^63^ This action is accompanied by inhibition of ERK1/2 phosphorylation, leading to a shift from propagation to cell death. Both actions were reversed by adding ion flux inhibitors, such as BaCl_2_ or amiloride, to prove dependence on ion flux. Prominently, drug inhibitors of p38β and p38α have the ability to delay tumor cell death, as did p38-specific siRNAs. Dissimilarly, healthy normal cells exhibited weaker response in ion flux and slight noticeable modifications in global transcription. Binding affinity for protein–lipid complexes clarifies the apparent assembly of their targets in cancer cells. Ho *et al.* conducted a proteomic analysis to identify such targets for 8000 human proteins.^67^ They recognized a huge number of proteins that bind to nucleotides as HAMLET complex targets. They identified 24 members of the Ras family of GTPases, 3 ATPases and 111 Kinases, representing whole divisions of the kinome tree. HAMLET complex revealed inhibition activity of Ras family and co-localization with GTPases in membrane clusters. Also, HAMLET complex was identified as a potent inhibitor of GTPases and kinases addicted to cancer cells. In addition, HAMLET complex has been identified as an inhibitor for kinase with a selectivity for tumor cells that spares normal cells. HAMLET complex performed as a pan-kinase inhibitor agent and decreased about 69% of kinase activity. These findings were confirmed using an antibody microarray to identify phosphorylated proteins in protein lysates of lung carcinoma cells after treatment with HAMLET.^67^

### c. Apoptotic action

Protein–lipid complexes exhibit toxicity and biological properties distinctive of the apoptosis process.^1,16,37,60,78–80^ The apoptotic action was essentially induced by the bound OA in the complex, showing that OA has potent effects on both the cellular membrane and mitochondria.^81^ Protein–lipid complexes show many signs of apoptotic effects on treated tumor cells, including caspase activation, cell shrinkage, DNA fragmentation and nuclear condensation.^1,7,13^ Moreover, the main apoptotic effects are on the mitochondrial inner membrane, opening the permeability transition pores and causing transfer of apoptogenic proteins from mitochondria into the cytoplasm.^82,83^ The BAMLET complex exerted apoptotic effects in several treated tumor cells by activating the caspase cascade. BAMLET complex also colocalizes with lysosomes in tumor cells, affecting the permeability of the lysosomal membrane and causing leakage of cathepsin L into the cytoplasm.^84^ Also, other apoptotic mechanisms related to the bound OA might be suggested, such as peroxisome proliferator activated receptor trigger, PKB pathway inhibition, the uncoupling of mitochondrial oxidative phosphorylation, stimulation of protein phosphatase type 2Cb, initiated production of active oxygen species, trigger of NF-κB stimulation, inhibition of tyrosine kinase and cell cycle arrest.^7,13,85^ The cell death enhancement by OA shows apoptotic characteristics in a caspase-3-independent manner in neuron cells,^85^ YAC-1 lymphoma cells^86^ and human leukemic HL-60 cells.^87^ It has been found that caspase-3-independent apoptosis and tyrosine kinase (TK)-inhibition were also induced after treatment by HAMLET and CAMLET, respectively.^7,60^ These findings evidenced that the bound OA in protein–lipid complexes might have a great effect in apoptosis and cell death through various mechanisms of effect.^88^ Fang *et al.* revealed that, although both free LF and LF–OA complex exhibited some similarities in terms of the apoptosis pathways regulated by certain expressed proteins, when the analogues in cell viability caused by LF–OA complex and free OA were taken into account, the apoptotic effects of LF–OA complex were more related to the OA than the proteinaceous shell.^89^

### d. Chromatin and histones interactions

Protein–lipid complexes have been found to interact with histones, chromatin and α-actinin, leading to perturbation of the chromatin structure and enhanced cell detachment.^62,66^ HAMLET complex showed the ability to enter the cytoplasmic membrane, cross the nuclear membrane, and accumulate in nuclei of cancer cells.^66^ Moreover, HAMLET complex rapidly internalizes in nuclei of tumor cells with 75% nuclear accumulation at 35 μM concentration of complex within only 1 h. However, in normal healthy cells, HAMLET complex has not shown any ability to translocate into the nuclei and only small quantities of the complex can enter healthy cell cytoplasm.^2,66^ This nuclear entry and accumulation of protein–lipid complexes was first confirmed through confocal microscopy using fluorophore-labeled streptavidin with biotinylated HAMLET and radiolabelled HAMLET; recently, both the α-LA and LF–OA complexes were traced by small angle neutron scattering using KWS2 spectrometry.^46,90^ HAMLET was shown to interact with other nuclear targets such as histones (H_2_B, H_3_ and H_4_), as confirmed by far western blot and MALDI-TOF analysis (mass spectrometry), and to interfere with nucleosome formation in the salt-jump technique.^91^ Additionally, HAMLET complex has been found to show activity in tumor cells by affecting chromatin acetylation, as in the synergistic effects of histone deacetylase inhibitors that change the accessibility of chromatin, promoting chromatin internalization of HAMLET and stimulating the tumoricidal effects of HAMLET complex, partly by activating the response of hyperacetylation.^46,92^ These findings propose that protein–lipid complexes ‘seal the fate’ of dying tumor cells *via* their strong affinity histone interferences and disruption of chromatin function and structure.^44^ Ho *et al.*^69^ traced 183 HAMLET-specific genes associated with transcription (39 genes), intracellular signaling cascades (25 genes), phosphate metabolic processes (20 genes), and cell cycle (21 genes) of HAMLET-treated cancer cells. Also, it was recently suggested that both alpha-lactalbumin- and lactoferrin–oleic acid complexes had effects on chromatin organization through compaction, which can lead to decrease in gene expression in the compacted chromatin regions and subsequently interfere with tumor cell functions. Using Ingenuity Pathway Analysis, they identified 131 cancer-related genes, half of which formed a cancer-related network, including NF_B1A, MAP3K8, SMAD7, and DUSP6.^69^

### e. Proteasome inhibition

In addition to perturbing tumor cell membranes with protein–lipid complexes and their accumulation in the nuclei of the tumor cells, this accumulation formed a misfolded protein-overload scenario that enhanced endoplasmic reticulum tension and allowed the complex to degrade the proteasomes. Proteasomes play an important role in normal cells by controlling the level of endogenous oxidized and misfolded proteins through extra-lysosomal degradation in the proteolytic barrel-shaped 20S proteasome core. Endogenous oxidized and misfolded proteins are degraded by both 26S and 20S proteasomes, while misfolded α-LA mainly interacts with barrel-shaped 20S proteasomes *in vitro*.^93,94^ HAMLET was found to co-localize with proteasomes in the cytoplasm and nuclei of tumor cells and exhibited inhibitory action of proteasome activity in entire tumor cell extracts.^44^ Remarkably, after incubation of intact 20S proteasomes with HAMLET complex *in vitro*, structural evidence for proteasome fragmentation by HAMLET complex was obtained.^44^ Gustafsson *et al.* demonstrated that HAMLET complex is targeted to the 20S proteasome core in tumor cells and modifies the proteasome structure with alterations in catalytic (β1 and β5) and conformational cores.^94^ Furthermore, protein–lipid complexes inhibit proteasome activity and fight degradation by proteasomal enzymes. Therefore, internalization of protein–lipid complexes to tumor cells, interference with proteasomes and perturbations of proteasome structure can combine with the cytotoxic activity of oxidized and misfolded protein complexes that attack the host cells.^95^

### f. Autophagic action

Autophagy (autophagocytosis) is a natural pathway of orderly lysosomal degradation and recycling that has been used by eukaryotic cells to regulate large protein aggregates, intracellular organelles and dysfunctional components that cannot be degraded by proteasome cores.^96,97^ In most eukaryotic cells, autophagy takes place at basal stages as an adaptive response in extreme cases such as starvation, which stimulates survival, but in other cases it appears to stimulate morbidity and cell death. Cells under starvation environments use cytoplasmic material again as a basal nutrients and consequently autophagy becomes an orderly adaptive stress response in dying cells to extend cell survival.^98,99^ It has been found that in parallel with the apoptosis process, protein–lipid complexes enhance autophagy, as confirmed by electron microscopy after treatment of tumor cells with HAMLET complex, which revealed that double membrane vesicles and typical modifications of macroautophagy, including translocation and accumulation of microtubule-associated proteins 1A/1B light chain 3 (LC3).^100^ Therefore, extreme responses cause a type II autophagic effect that leads to cell death.^101^ Moreover, inhibition of autophagy by Atg5 and Beclin-1 considerably decreased the HAMLET-induced tumor cell death, demonstrating that both Beclin-1 and Atg5 were involved in the process of autophagosome formation. It has been generally established that autophagy seems to be a cellular mechanism playing a crucial role in HAMLET-induced cell death.^101^ The findings achieved using protein–lipid complex treatment can be associated to those achieved in determining the effect of unsaturated fatty acids in apoptosis and autophagy in hepatic tumor cells, since free OA has been found to induce autophagy in hepatoma (HepG-2) cells *in vitro*.^102^ One study reported that the cytotoxicity of protein–lipid complexes other than HAMLET seemed to include neither an autophagy mechanism nor a classic apoptosis pathway, but these active complexes have tumoricidal effect through a mechanism including leakage of cathepsins after perturbing the lysosomal membrane.^84^ Definitely, unsaturated fatty acids like OA are membrane permeabilization enhancers *via* increasing the structural flexibility of lipid bilayers, consequently creating leakage of cellular fluids or perturbing effects on lipid bilayers. In addition, HAMLET complex interferes with lipid bilayers, disrupting their integrity and structure, and this interference involves the contribution of the fatty acid component.^32^

### g. Cell detachment action

Cell adhesion is an essential process for tissue integrity, in which cells attach and interact to neighbouring cells through particular interactions of the cell surface molecules. The capability of protein–lipid complexes to enhance adherent tumor cell detachment has been described in response to treatment both *in vitro* and *in vivo*.^7,13,103,104^ Affecting the cell detachment process with extracellular components strongly changes cellular survival and propagation. Tumor cells have the capability to proliferate in a cell anchorage manner and cell detachment from the location of the main tumor may establish the first stage in tissue homeostasis and disease.^105^ Cell detachment requires component alterations in the exact intercellular adhesion components by molecular interference, including significant complexes of the cytoskeleton like α-actinin. Alpha-actinin arises in the areas of connection between membranes and actin filaments and seems to play a strong role in the movement of integral membrane proteins and in anchoring microfilament bundles to membranes.^106,107^ Alpha-actinin interferes with model membranes, comprised of fatty acids and glycerides, and unsaturated fatty acids affect the structural organization and regulation of the cytoskeleton.^108–110^ Also, α-actinins are crosslinking proteins and the main F-actin binders in human cells. HAMLET complex definitely exhibits interference with α-actinin and enhances the detachment process. To recognize the components integrated into the detachment process, tumor cellular extracts were analyzed after treatment with HAMLET complex. Both α-actinin-1 and -4 were identified as binding targets for HAMLET complex.^104^ The cellular sites of α-actinins vary depending on the cell type and both α-actinin-1 and -4 form functional antiparallel homodimers.^111,112^ Alpha-actinin-1 localizes at adherence connections or at focal adhesion plaques, whereas α-actinin-4 has been shown to localize at sites of cell–cell connection. Moreover, α-actinin-4 may also interfere with focal adhesion ingredients, such as vinculin and cytoplasmic β-integrins.^107,113,114^ Interactions of biotinylated α-actinin-4 peptides with HAMLET complex were quantified by a library map of synthetic peptides using a cryoelectron microscopy reconstruction illustration of smooth muscle α-actinin.^104^ The four peptides opposite the cavity between the central rod domain and the actin-binding domain were identified, developing a strong joined binding location for HAMLET complex.^62^ Moreover, a potential β-integrin-binding location was recognized and the HAMLET complex interacted with two peptides in the C-terminal of α-actinin. HAMLET complex is associated with signalling molecules, ion channels, rapid perturbation of the focal adhesion components cytoplasmic domains of transmembrane receptors, cytoskeletal structure, signalling, and focal adhesion kinase phosphorylation. HAMLET complex exerted its effects only on tumor cells, sparing normal differentiated cells, which retained their integrity.^62^ In addition, an *in vivo* investigation into the effect of HAMLET complex on bladder cancer was concluded. HAMLET complex has been shown to trigger enormous detaching of dead tumor cells into the urine, demonstrating that the protein–lipid complexes may display cell detachment properties towards solid cancers.^4^

### h. Caspase cascade and Bcl-2 mRNAs activation

HAMLET complex showed, in early studies, its capability to co-localize in mitochondria of tumor cells, which indicates interaction with mitochondrial proteins of tumor cells. This affinity of the HAMLET complex was determined using extracted mitochondria, where HAMLET complex enhanced the depolarization of the lipid bilayers, causing leakage of cytochrome c.^57^ The variation in sensitivity to protein–lipid complex treatment between sensitive and resistant cells is interesting. HAMLET complex has been shown to trigger pro-apoptotic caspase cascade involving caspase 3 and caspase 6, but HAMLET's tumoricidal activity did not depend on caspases only, as some caspase inhibitors did not inhibit apoptosis and some cell lines lacking caspase 3 did not demonstrate resistance to HAMLET-induced apoptosis. Consistent with these findings, caspase inhibitors, such as ZVAD, did not prevent HAMLET complex from internalizing from the cellular cytoplasm to the nuclei of tumor cells.^79^ Caspases are triggered in tumor cells that die after exposure to protein–lipid complexes, but caspase inhibitors do not prevent the treated cells from death. The main role of caspases as stimulators of HAMLET-induced apoptosis remains unclear. Discovery of the principal mechanisms might clarify nearby essential cellular developments that differentiate tumor cells from healthy cells. Svensson *et al.* demonstrated that both caspase and Bcl-2 family mRNAs appeared in both sensitive and resistant cells before and after treatment with HAMLET complex using RNA protection techniques.^78^ Their findings exhibited a change in the profile of Bcl-2 family mRNA between tumor cells and normal cells with different sensitivities to treatment, but no variance in the responses to HAMLET treatment between the two cell types with the regulation of Bcl-2 marker in HAMLET-exposed cells.^80^ A related method was used to determine the specificity of mRNAs for various caspases. The authors demonstrated that there was a change in profile of caspase mRNA between the two cell types, but not in the levels of caspase mRNA, excepting caspase 2 in one cell type and a 2-fold increase in caspase 9 in another cell line. Also, these trials exhibited no clear mechanism linking specific caspase mRNA species to HAMLET sensitivity. Moreover, both BAMLET complex and LF–OA complex have been suggested to enhance cell death through the induction of a caspase cascade mechanism.^12,84^

### i. Anti-glycolysis action

Most tumor cells show a rise in the glycolysis pathway and utilize this metabolic mechanism for ATP generation as a fundamental basis of their energy source.^115,116^ This phenomenon is considered one of the major essential metabolic modifications of malignant development and is named the Warburg effect.^116,117^ Increased glycolysis pathway has been observed in numerous tumor cells, suggesting that this metabolic modification is common to most tumor cells.^116,118^ Tumor therapy scenarios would consider metabolism variations between normal and tumor cells and, consequently, prevention of the glycolysis pathway will probably affect the proliferation of tumor cells and therefore decrease cancer development.^119,120^ Wide studies have been conducted with the target of recognizing the modes of glycolysis and glutaminolysis in tumor cells and improving appropriate therapeutic efficacy for tumor treatment.^118,120^ Some glycolytic inhibitors, such as 2-deoxyglucose (glucose analog) and 3-bromopyruvate (hexokinase inhibitor), have demonstrated a potent effect in reducing the proliferation of numerous types of tumor cells.^118,121,122^ Interestingly, fatty acids have been found to be effective chemotherapeutic agents against adjunctive colorectal cancer.^121^ To understand the molecular scenarios of the antiproliferative mechanism of protein–lipid complexes, an extensive study demonstrated the action of HAMLET complex on A549 lung carcinoma cells, using a combination of proteomic, metabolomic and genetic technologies to identify modified cell metabolisms and properties of protein interaction.^65^ The authors demonstrated that HAMLET treatment affected the glycolysis-related proteins and oncogene c-Myc marker. They also concluded that HAMLET has the ability to modify cell metabolism and perturb tumor cell glycolysis through inhibiting hexokinase I. These findings were confirmed by confocal microscopy and functional protein array techniques; it was also revealed that HAMLET complex interacts with hexokinase I in A549 cell line. Tumor cells treated with HAMLET complex showed a marked 120-fold increase of OA concentration using GC-MS profiling of cellular metabolites. Also, other metabolites related to the glycolysis process were less abundant, in line with the HAMLET-mediated inhibition of glycolysis.^65,123^ Generally, this anti-glycolysis can be attributed to the action of the OA part alone and is not fundamentally linked with its proteinaceous compartment in the complex. Definitely, glycolysis inhibition is already a potent scenario effectively used for tumor therapy^118^ and OA is a potent inhibitor of numerous glycolytic enzymes.^124^ Free OA was found to inhibit hexokinase I,^125^ whose function is converting glucose into glucose-6-phosphate in the glycolysis cycle.^126,127^ Hence, utilizing protein–lipid complexes like HAMLET as a carriers of fatty acids like OA might be beneficial to modify tumor cell metabolism and perturb the glycolysis cycle, which is stimulated in tumor cells owing to the Warburg effect as mentioned above.

## Other biological activities of the protein–OA complexes

**Fig. 5:**
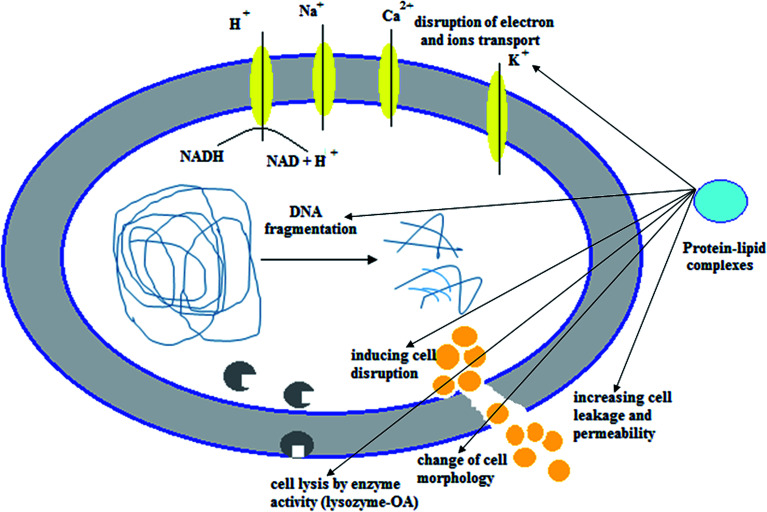
Overview of the cytotoxic protein–lipid complexes' activities against Gram negative bacteria.

Antitumor efficacy of HAMLET complex was revealed initially during attempts to examine the potent anti-adhesive activity of human milk casein fractions on bacterial binding to alveolar lung tumor cells. Human casein also had the ability to reduce the attachment of bacterium *Streptococcus pneumoniae* to respiratory tract cells.^29^ Therefore, the authors used different fractions from milk casein in more studies to understand the molecular mode and the specificity included in host cell recognition by pneumococci.^128^ Besides the tumoricidal activity of HAMLET and HAMLET-like complexes, they exhibited significant antibacterial activity against many kinds of bacteria (Fig. 5).^63,129–133^ HAMLET was found to kill certain bacterial strains, such as *S. pneumonia, Haemophilus influenzae*, antibiotic-susceptible and -resistant *Streptococcus pyogenes* (GAS) and *Streptococcus agalactiae* (GBS), and multidrug-resistant *Mycobacterium tuberculosis* (MD-TB),^128,133,134^ and sub-toxic doses of HAMLET not only stimulate the bactericidal effect for treatment of pneumococci,^135^ but potentiate a remarkably broad array of TB drugs and antibiotics against *M. tuberculosis* as well as *S. pneumoniae*, GAS and GBS. The synergistic action of HAMLET on the viability of TB decreased the minimal inhibitory concentrations of rifampin, bedaquiline, delamanid, and clarithromycin by 8- to 16-fold. HAMLET also killed *M. tuberculosis* and enhanced the efficacy of TB drugs inside macrophages and in the natural habitat of *M. tuberculosis*.^133^ Moreover, the combination of HAMLET complex and antibiotics (gentamicin, erythromycin, and penicillin) decreased the doses of antibiotics required and increased the sensitivity of both sensitive and multi-antibiotic-resistant bacteria.^135,136^ Analysis of the antibacterial spectrum indicated that most Gram-negative bacteria were found to be sensitive to treatment with HAMLET complex while other Gram-positive bacteria were resistant. Numerous phenotypes observed during HAMLET-caused tumor cell death were also detected in bacteria, implying that a similar apoptosis response is triggered by HAMLET in antibacterial mechanisms.^135,137^ Remarkable similarities were detected from use of HAMLET, including an alteration in morphology of bacterial cells and DNA fragmentation.^134^ As expected, the antibacterial effect of HAMLET is also attributed to the OA component, since, among the diverse biological activities of long-chain fatty acids, they have potent efficacy to inhibit bacterial growth as well as to kill the bacteria.^138,139^ HAMLET complex was found to induce Ca^2+^-dependent membrane depolarization and membrane permeability and death in bacteria in similarity with host cells (Fig. 5).^29,133,134,140^ Mycobacteria have the lowest known cell permeability among bacteria,^141^ while Gram-positive bacteria such as *S. pneumoniae* have a rather high cell permeability.^142^ By a similar mechanism, HAMLET effectively killed many bacterial pathogens, possibly by calcium and/or sodium transport inhibition.^140^ Furthermore, other bioactive protein–OA complexes, such as bovine α-LA–OA complex, pike parvalbumin–OA (bLG–OA-45 and pPA–OA-45) and equine lysozyme, were found also to induce *S. pneumoniae* D39 cell apoptosis leading to cell death, with a mechanism analogous to those for HAMLET complex.^25,131^ The authors showed that the cytotoxic activity of these active complexes improved with increasing OA content in the mixtures, which induced damage and depolarized the plasma membrane of *S. pneumonia* D39 after treatment. This may prove the importance of the lipidomic part in these complexes and other like-complexes in bacterial viability.^143,144^ Moreover, the therapeutic efficacy of HAMLET complex against human skin papillomas was demonstrated by Gustafsson *et al.*^3^ HAMLET complex showed significant efficacy in patients with observed severe treatment-resistant papilloma disease on feet and hands after topical treatment once a day for 3 weeks with saline solution as control. HAMLET complex had the ability to reduce the lesion size by greater than 75%. Additionally, a potent reduction in lesion size was detected in all cases treated with HAMLET complex and all lesions had completely resolved in 83% of the patients treated for 48 months.

## Conclusion and future perspectives of protein–lipid complexes

It is very intriguing to discover potent and differential tumoricidal activity from two natural components of breast milk and other sources without direct association to milk. The partly unfolded conformation of α-LA can form a cytotoxic complex with OA, where the complex exerts selective cell death action specific to tumor cells both *in vitro* and *in vivo*. This partially unfolded human α-LA in the apo-state (α-LA after removing calcium ion) is capable of interacting with OA to form the cytotoxic complex called HAMLET. *In vitro* selectivity and promising *in vivo* findings of protein–lipid complexes make this area of exploration very fascinating. Obviously, there are various questions to be answered. The exact molecular mechanisms and scenarios of apoptosis and cell death induced by protein–lipid complexes are not sufficiently understood. Numerous scenarios and cellular targets have been proposed to explain the tumoricidal effect of HAMLET and HAMLET-like complexes. There are two different types of effects for these complexes, membrane perturbation and accumulation in the cytoplasm and nucleus of the tumor cell. Perturbation of cellular membranes is the first action and intracellular actions, such as change of proteasome and chromatin structures and caspase stimulation, are secondary. Some authors have proposed that the tumoricidal activity of the formed active protein–lipid complexes is attributed to the OA component in the complex, which is considered the main player in the stimulation of tumor cell death. According to various studies, protein–lipid complexes killed cancer cells and undifferentiated cells, sparing normal healthy cells and the area surrounding solid tumors. Interference of HAMLET complex with α-actinin and proteasome might be related to the action demonstrated for free OA alone. Moreover, other biological functions of HAMLET complex, such as the bactericidal effect, might be similar to that of OA since OA has potent bactericidal action on *Streptococci* group A bacteria and showed antibacterial and antibiofilm effects against *Staphylococcus aureus*. On the other hand, other reports have suggested that the action of cytotoxic protein–lipid complexes other than HAMLET and BAMLET might be attributed to the synergistic effect of both OA and protein parts. Such synergistic tumoricidal effects will be different from one protein to another. Active protein–lipid complexes can be prepared using various approaches and their activity scenarios seem to be different. For instance, the internalization of HAMLET complex occurs directly, as investigated in many reports, while the uptake of lysozyme–OA complex by tumor cells happens after perturbing the cellular membrane, with consequent accumulation inside the cells. Furthermore, cell death enhanced by HAMLET or BAMLET is different, according to their co-localization in lysosomes, mitochondria and the nuclei of tumor cells, lysosomal death effect and the action of the autophagy process.

The efficacy of protein–lipid complexes as therapeutic candidates has been examined in various models *in vivo*. These models including skin papillomas, patients with bladder cancer, glioblastoma rat model, and MRSA-infected rat model. All trials indicated that HAMLET complex could decrease the tumor volume or lead to healing from the infectious disease without any cytotoxic effect to the surrounding normal area. These findings propose that infectious pathogens and tumor cells are more sensitive to HAMLET compared with the normal cells. Recently, new results on the preparation of protein–lipid complexes and their biological roles are rapidly being discovered. Indeed, there is a crucial requirement to identify whether the structural and molecular features of the cytotoxic protein–lipid complexes correlate with their new biological efficiency. This requires comparing the tumoricidal efficacies of different active protein–lipid complexes while changing protein/fatty acid ratios and the size range, as well as a determination whether these structures are retained upon uptake to cellular circumstances. Also, the fate of the fatty acid, which seems to be released once the protein–lipid micelles are added to the cellular membrane, must be discovered. The development of these new protein–lipid complexes will definitely persuade further study into the structural forms that proteins may adopt under various environments, thus eventually increasing our overall knowledge of protein characteristics. Protein–lipid complexes should be explored in controlled trials of skin papillomas, brain tumors, and bladder cancer, where human and experimental data are accessible. Supplementary characterization of primary molecular actions of cell death regulation would be suitable to propose forthcoming disease remedies including both prokaryotic and eukaryotic cells.

Furthermore, there are many fatty acids other than OA with more stability to metabolic hydrolysis, such as Minerval® (2-hydroxyoleic fatty acid).^145,146^ Minerval® is considered a potent therapeutic agent for targeting the cellular membrane and is supposed to accomplish its tumoricidal action by modifying the biophysical features of lipid bilayers, leading to activation and modification of cellular signaling and amphitropic proteins and finally causing cell death.^147^ Therefore, it appears reasonable to suggest preparation of new protein–lipid complexes with Minerval® and investigation of their tumoricidal and other biological effects. Finally, there are numerous dairy products containing very important proteins present in high concentrations with potent biological roles that can be produced using various simple techniques, such as heat treatment, acid pH, and pressure. These proteins also can be converted to partially unfolded forms which make the protein ready to bind OA using simple approaches.

## Conflicts of interest

The authors declare that there is no conflict of interest regarding the publication of this manuscript.

## Supplementary Material
